# Perceived coping & concern predict terrorism preparedness in Australia

**DOI:** 10.1186/1471-2458-12-1117

**Published:** 2012-12-27

**Authors:** Garry Stevens, Kingsley Agho, Melanie Taylor, Alison L Jones, Margo Barr, Beverley Raphael

**Affiliations:** 1 University of Western Sydney, School of Medicine, Building EV, Parramatta Campus, Locked Bag 1797, Penrith, NSW 2751, Australia; 2 University of Wollongong, Graduate School of Medicine, Building 28, Wollongong, NSW 2522, Australia; 3 Illawarra Health and Medical Research Institute, Wollongong, NSW, Australia; 4 Centre for Epidemiology and Research, New South Wales Ministry of Health, 73 Miller Street, North Sydney, NSW 2060, Australia

**Keywords:** Terrorism, Resilience, Coping, Self-efficacy, Preparedness, Avoidance, Behaviours

## Abstract

**Background:**

In the aftermath of major terrorist incidents research shows population shifts towards protective behaviours, including specific preparedness and avoidance responses. Less is known about individual preparedness in populations with high assumed threat but limited direct exposure, such as Australia. In this study we aimed to determine whether individuals with high perceived coping and higher concern would show greater preparedness to respond to terrorism threats.

**Methods:**

Adults in New South Wales (NSW) completed terrorism perception and response questions as part of computer assisted telephone interviews (CATI) in 2010 (N=2038). Responses were weighted against the NSW population. Multiple logistic regression analyses were conducted to evaluate the relationship between personal coping/concern factors and terrorism-related preparedness and avoidance behaviours, and to control for potential confounders such as socio-demographic and threat perception factors.

**Results:**

Increased vigilance for suspicious behaviours was the most commonly reported behavioural response to perceived terrorism threat. Multivariate analyses showed that the factor combination of high perceived coping and higher concern was the most consistent predictor of terrorism preparedness behaviours and evacuation intentions, including increased vigilance (Adjusted Odd Ratios (AOR)=2.07, p=0.001) learning evacuation plans (AOR=1.61, p=0.05), establishing emergency contact plans (AOR=2.73, p<0.001), willingness to evacuate homes (AOR=2.20, p=0.039), and willingness to evacuate workplaces or public facilities (AOR=6.19, p=0.015) during potential future incidents.

**Conclusion:**

The findings of this study suggest that terrorism preparedness behaviours are strongly associated with perceived high coping but that this relationship is also mediated by personal concerns relating to this threat. Cognitive variables such as coping self-efficacy are increasingly targeted as part of natural hazard preparedness and are a viable intervention target for terrorism preparedness initiatives. Raising individual coping perceptions may promote greater general and incident-specific preparedness and could form an integral element of community resilience strategies regarding this threat.

## Background

While levels of community distress and anxiety typically decline in the months following major terrorist attacks, population shifts towards ‘protective’ behaviours may persist for longer periods [1,2]. Such changes include altered use of public transport systems and air travel, avoidance of places of perceived high risk and increased substance abuse [2-5]. Collectively, such behaviours may have substantial economic and health impacts. Significantly reduced travel was observed on the London underground 12 months after the 2005 London transport bombing, while reductions in U.S. air travel continued up to two years after the 9/11 attacks [5,6]. Paradoxically, this latter shift towards presumed ‘safer’ travel resulted in an estimated 1500 additional road fatalities in the first year following the attacks [7].

Previous research regarding coping responses to terrorism threat has examined ‘problem-focused’ coping, which includes preparedness activities to address specific issues (e.g. learning evacuation plans, establishing emergency contact plans) and ‘emotion-focused’ coping which aims to manage the associated stress [8]. More critical issues may relate to whether such responses are primarily cognitive or behavioural in nature and particularly whether they constitute avoidance coping [4,9,10]. There is evidence that terrorism preparedness and avoidance represent qualitatively distinct factors [9] and are associated with adaptive and maladaptive outcomes respectively. For example, preparedness responses after the 9/11 attacks were associated with lower psychological distress, while forms of mental and behavioural avoidance predicted higher distress and psychopathology [11,12]. Similar findings have been observed in Canada, despite its history of limited direct exposure [9].

Behavioural responses to terrorism threat are also associated with specific cognitive, affective and demographic factors. Threat appraisal models show that such responses depend on our judgement of specific threat elements (i.e. perceived likelihood, seriousness) but are often more influenced by our perceived ability to cope with them [8,13]. Coping perceptions or ‘self efficacy’ has been shown to be one of the strongest cognitive predictors of terrorism preparedness behaviours [14], while affective states such as worry and concern are associated with both preparedness and avoidance [14,15]. Despite such findings, few studies have simultaneously examined concern and perceived efficacy as predictors of terrorism preparedness in the general community [14]. This is notable in that efficacy perceptions are increasingly targeted as part of natural hazard preparedness [16] and are key predictors of health worker willingness to respond to terrorist incidents [17,18]. Further research is needed regarding the role of these factors in individual preparedness for terrorism, as this may critically inform preparedness initiatives for the general population.

Research examining community preparedness also needs to consider such responses across a range of terrorism event phases. While avoidance and preparedness may be high following an attack, information is also required about response factors in situations of perceived pending threat [9]. Australia’s recent history of limited direct exposure but high assumed threat [19] provides a suitable population for such research. The aim of this study was to determine whether high perceived coping in relation to terrorism and concurrent high concern are associated with terrorism preparedness in Australia.

## Methods

A search of existing survey instruments was conducted to identify items that would address the study aim and be most relevant to the Australian threat context. The terrorism question module consisted of validated and reliable items adopted from two previous surveys [3,10]. These assessed: perceived likelihood of terrorism; concerns for self/family; perceived ability to cope in the event of a terrorist attack; emergency evacuation intentions; and current avoidance/preparedness behaviours. For the latter, a prologue to the question set framed a general response period and threat examples: “In recent years terrorist attacks, such as bombings and shootings, have occurred in a number of countries.” All study protocols and procedures were approved by the New South Wales Ministry of Health and the University of Western Sydney ethics committee (protocol no. H7143).

### Outcome variables

#### Behavioural responses

Individual responses to the threat of terrorism were assessed with the primary question: “How much have you done any of the following due to the risk of a terrorist attack.” This was followed by six current preparedness and avoidance behaviours, and two hypothetical items assessing evacuation intentions. The three avoidance items were: “avoided certain public places e.g. central business district, national monuments or crowd events”; “changed use of public transport” and “changed or delayed plans for an overseas holiday”. The latter item was chosen as it represented discretionary air travel, which was potentially more sensitive to perceived regional or international threats [3].

The terrorism preparedness questions were: “learned the evacuation plan of a building you occupy frequently”; “made an emergency family contact plan” and “became more aware of suspicious behaviour of others” (hereafter ‘vigilance’). Separate items assessed willingness to evacuate from either a home or a workplace/public facility in the context of a potential terrorist incident.

Responses to all eight behaviour questions were rated on a five-point Likert scale (1–5: ‘not at all’, ‘a little’, ‘moderately’, ‘very’ and ‘extremely’). Other possible responses were ‘don’t know’, refused and ‘not applicable’ (e.g. effectively never used public transport). For statistical purposes response categories were dichotomised to form the outcome variables of interest (e.g. changed use of public transport), with a value ‘1’ assigned to a response of ‘moderately’, ‘very’ or ‘extremely’ and ‘0’ to all other responses. This method was used for all variables except for the two evacuation intention items. As evacuation intentions in this context are generally quite high and to be consistent with previous treatment, [19] ‘very much’ and ‘extremely’ were combined into the indicator of interest; ‘willingness to evacuate’.

### Predictor variables

#### Terrorism concern and coping perceptions

To address the study aim a combined variable of terrorism-related concern and perceived coping was included in the analysis to evaluate its relationship with preparedness behaviours. Concern and coping were each measured with a single item: “If a terrorist attack happened in Australia, how concerned are you that you or your family would be directly affected?” and “If you were in an area affected by a terrorist attack, how well do you think you would be able to cope in that situation?”. These items were also scored as noted above to represent ‘low’ and ‘high’ response categories (e.g. high concern). High concern/high coping represented the independent variable of primary interest, with recent evidence suggesting that this factor combination may be the most consistent predictor of preparedness behaviours [14,17].

### Potential confounding factors

#### Demographic and health variables

The socio-demographic and health factors examined for their associations with behavioural responses were: age; highest educational qualification; pre-tax household income; marital status; number of children ≤ 16 years of age in household; residential location (urban or rural, as determined by Area Health authority); location remoteness as determined by Accessibility/Remoteness Index of Australia (ARIA+); being born in Australia; ethnic minority status (i.e. speak a language other than English at home); employment status; and self-rated health status.

#### Cognitive and affective variables

Additional cognitive variables consisted of an item assessing perceived terrorism likelihood in Australia and a general measure of perceived personal resilience, the abbreviated Connor-Davidson Resilience Scale (CD-RISC2). This scale uses two items which separately measure personal adaptability and ability to continue to function effectively in stressful circumstances. Based on established community norms, summed scores of 0–6 and 7–8 represent low and high self-rated personal resilience, respectively [20]. Current psychological distress was measured using the 10-item Kessler Psychological Distress Scale (K10). Scores on the K10 range from 10–50, with ≥ 22 being considered ‘high’ psychological distress [21].

### Administration

The terrorism question module was administered as part of a wider survey examining a range of potential threats (e.g. pandemic influenza, climate change). It was administered at the NSW Health Survey program. The survey was conducted between 29 October 2009 and 20 February 2010 using the established Computer Assisted Telephone Interview (CATI) and sampling methodology of the NSW Ministry of Health [22]. The target population for the survey was all residents aged 16 years and over, living in NSW and stratified by the state’s eight area health services. Quota sampling was used to ensure geographic representation by respective urban/rural area health services. Location remoteness (ARIA+) was classified post hoc based on respondent address post code. Households were contacted using random digit dialling (RDD) and the survey was conducted in English. Residential phone numbers were used in the sample, as residential phone coverage remains relatively high in Australia at approximately 85% of all households, [23] and the proportion of mobile phone only households was regarded as sufficiently low at the time of survey (8.7%) as to have a low impact on health estimates obtained using this method [24]. Up to 7 calls were made to establish initial contact with a household, and up to 5 further calls to contact a selected respondent. Potential respondents were identified according to the closest upcoming birthday within the household to ensure random selection. Verbal consent to participate was obtained prior to survey commencement and followed detailing of survey aims and content.

### Statistical analysis

The survey data were weighted to be representative of the target population and to adjust for probability of selection and differing response rates among males and females and different age groups. This was done in accordance with NSW Health Population Health Survey Program weighting methodology [25]. This weighting strategy also means that these data are representative of the national population in terms of its key demographic characteristics [26]. Data analysis was performed using the “SVY” commands of Stata version 12.0 (Stata Corp, College Station, TX, USA), which allowed for adjustments for sampling weights. The Taylor series linearization method was used in the survey when estimating confidence intervals around prevalence estimates.

Multiple logistic regression analysis was conducted using a backward stepwise method in order to determine the independent variables (socio-demographic, threat perception and concern/coping factors) significantly associated with current terrorism-related behaviours and evacuation intentions. The models were constructed by backward elimination and used the following procedures: 1) only variables with p-value < 0.20 in the Univariate analysis were entered into the models for backward elimination; 2) the screened variables (potential confounders) were included in the model and the non-significant variables (p > 0.05) were manually eliminated step by step and 3) any variables removed from the final regression model due to collinearity were reported. The main predictor variable (combined concern/coping) was retained in all the final models. The odds ratios with 95% CIs were calculated in order to assess the adjusted risk of the independent variables. Those with p < 0.05 were retained in the final model.

## Results

A total of 2,038 state residents completed the terrorism survey module. The survey response rate was determined in accordance with NSW Health Survey methodology and calculated as total completed interviews of eligible participants, divided by the combined total of completed interviews and refusals [22]. The survey process yielded 3548 eligible participants (2,038 completions and 1510 refusals) and a final response rate of 57.4%.

The prevalence estimates regarding avoidance and preparedness behaviours and evacuation intentions are presented in Table 1. With regard to terrorism-related avoidance, 13.7% reported (moderate to extreme) avoidance of some public places or events, 5.6% reported changes in their use of public transport and 20.4% had deferred or changed plans for overseas holiday travel. With regard to terrorism-related preparedness, 45.0% reported moderate to extreme increases in their vigilance for suspicious behaviours/activities, 39.0% had learned the evacuation plan of a building they occupy frequently and 19.7% developed an emergency family contact plan. In relation to evacuation intentions, 74.9% reported being very/extremely willing to evacuate from their home in the context of a possible terrorist threat and 88.0% reported being very/extremely willing to evacuate from a workplace/public facility in such circumstances.

**Table 1:** Prevalence estimates for current terrorism-related avoidance and preparedness behaviours and evacuation intentions

**Question**	**Response**	**%**	**95% LCI**	**95% UCI**
**Avoid certain places (CBD, national monuments, crowd events)**	Not at all	76.91	74.11	79.48
	A little	8.252	6.643	10.21
	Moderately	7.606	6.173	9.339
	Very	3.407	2.201	5.239
	Extremely	2.76	2.038	3.728
	Don’t know / N/A	0.0346	0.0086	0.1391
	Refused	1.035	0.6482	1.65
**Changed use of public transport**	Not at all	52.67	49.48	55.84
	A little	4.155	2.97	5.786
	Moderately	2.664	1.851	3.821
	Very	2.036	1.126	3.654
	Extremely	1.024	0.6559	1.596
	Don’t know / N/A	37.01	34.09	40.02
	Refused	0.44	0.17	1.08
**Changed plans for overseas travel**	Not at all	53.8	50.61	56.96
	A little	10.09	8.185	12.37
	Moderately	9.366	7.827	11.17
	Very	6.025	4.497	8.028
	Extremely	5.027	3.98	6.332
	Don’t know / N/A	15.26	13.39	17.33
	Refused	0.4358	0.2237	0.8475
**More vigilant for suspicious behaviours**	Not at all	27.37	24.61	30.32
	A little	27.12	24.27	30.17
	Moderately	26.1	23.33	29.06
	Very	11.51	9.708	13.6
	Extremely	7.412	6.088	8.998
	Don’t know / N/A	0.0212	0.003	0.1509
	Refused	0.4715	0.2757	0.8054
**Learned evacuation plan of frequently occupied building**	Not at all	23.98	21.35	26.82
	A little	10.53	8.398	13.12
	Moderately	16.37	13.91	19.18
	Very	9.335	7.636	11.37
	Extremely	13.39	11.38	15.71
	Don’t know / N/A	26.04	23.61	28.62
	Refused	0.3497	0.1779	0.6861
**Made emergency family contact plan**	Not at all	72.56	69.73	75.22
	A little	6.243	4.917	7.898
	Moderately	9.983	8.32	11.94
	Very	3.923	2.914	5.263
	Extremely	5.778	4.459	7.456
	Don’t know / N/A	1.199	0.813	1.766
	Refused	0.3143	0.1644	0.6001
**Willing to evacuate home**	Not at all	4.611	3.623	5.851
	A little	4.89	3.649	6.525
	Moderately	14.68	12.49	17.18
	Very	24.88	22.15	27.82
	Extremely	50.1	46.89	53.31
	Don’t know / N/A	0.0294	0.0041	0.2086
	Refused	0.8114	0.4968	1.323
**Willing to evacuate workplace/public facility**	Not at all	1.021	0.6661	1.562
	A little	1.603	0.9955	2.573
	Moderately	8.595	6.88	10.69
	Very	25.82	23.08	28.77
	Extremely	62.2	59.02	65.27
	Don’t know / N/A	0.7615	0.5097	1.136
	Refused	0	0	0

1. Lower confidence interval (LCI), upper confidence interval (UCI).

2. N/A – not applicable to respondent circumstances.

### Avoidance

The results of the multivariate analysis for the terrorism-related avoidance behaviours are presented in Table 2. Respondents with middle high school qualifications were significantly more likely to report avoidance of places/events than those with university qualifications (Adjusted Odd Ratios (AOR)=2.22, p=0.002). When perceived terrorism likelihood replaced educational qualification in the final model, those who perceived an attack as more likely were also significantly more likely to report avoidance of places or events (AOR=1.45, p=0.047).

**Table 2:** Terrorism avoidance behaviours by socio-demographic & threat perception variables - adjusted odds ratios (AOR)

**Outcome variable**	**Independent variable**	**Adjusted odd ratios**		
		**AOR**	**[95% CI]**	***p***
**Avoid certain places**				
	**Highest qualification**			
	University degree	1.00		
	Vocational college diploma	1.21	(0.70, 2.08)	0.501
	High school certificate	0.74	(0.37, 1.48)	0.389
	Middle high school certificate	**2.22**	**(1.34, 3.68)**	**0.002**
	None	1.30	(0.60, 2.82)	0.512
**Changed use public transport**				
	**Residential location (ARIA+)**			
	Highly accessible (urban)	1.00		
	Accessible	0.92	(0.45, 1.85)	0.810
	Moderately accessible	**0.38**	**(0.15, 0.96)**	**0.040**
	Remote/Very remote	0.16	(0.02, 1.30)	0.086
	**Household income ($A)**			
	<$20 k	1.00		
	$20-40 k	**2.48**	**(1.07, 5.71)**	**0.033**
	$40-60 k	1.07	(0.38, 3.03)	0.892
	$60-80 k	1.03	(0.38, 2.82)	0.955
	>$80 k	0.65	(0.27, 1.55)	0.333
**Changed plans overseas travel**				
	**Terrorist attack likely**			
	No	1.00		
	Yes	**1.57**	**(1.13, 2.17)**	**0.007**
	**High psychological distress**			
	No	1.00		
	Yes	**1.55**	**(1.00, 2.39)**	**0.048**
	**Household income ($A)**			
	<$20 k	1.00		
	$20-40 k	**1.86**	**(1.02, 3.39)**	**0.042**
	$40-60 k	**1.91**	**(1.03, 3.54)**	**0.040**
	$60-80 k	**1.89**	**(1.00, 3.56)**	**0.049**
	>$80 k	1.75	(0.99, 3.08)	0.053

1. Note: 95% confidence intervals (CI) that include 1.00 indicate a non significant result.

2. Independent variables controlled for were: age; highest educational qualification; household income, no. of children ≤ 16 years in household; residential location (urban or rural, and location remoteness via Accessibility/Remoteness Index of Australia (ARIA+); being born in Australia; speaking a language other than English at home (‘minority status’); perceived likelihood of terrorism and self-rated health status, personal resilience (CD-RISC2) and psychological distress (K10).

3. Psychological distress was measured using the K10. Values range from 10–50, with ≥22 considered ‘high’ psychological distress.

Respondents living in urban (highly accessible) areas were significantly more likely to have changed their use of public transport, compared to those in rural (moderately accessible) areas (AOR=0.38, p=0.040), as were those with low household incomes ($20,000-$40,000) compared to those on very low household incomes (≤ $20,000) (AOR=2.48, p=0.033). Higher likelihood of having changed or deferred overseas travel due to terrorism concerns was associated with: high perceived likelihood of terrorism within Australia (AOR=1.57, p=0.007); high psychological distress (AOR=1.55, p=0.048); and middle range ($40,000-$60,000) compared to very low household incomes (<$20,000) (AOR=1.91, p=0.04).

**Figure 1:**
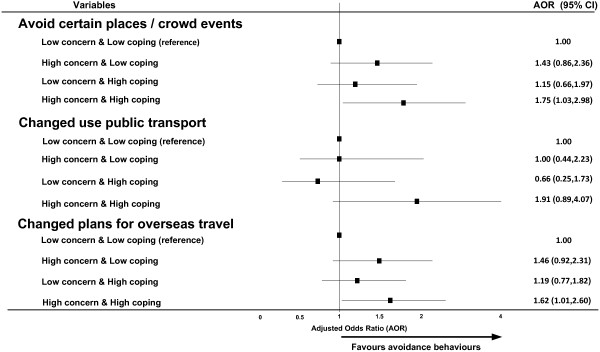
Terrorism related avoidance behaviours by concern/coping combined indicator.

Figure 1 presents the adjusted odds ratios (AOR) of the combined concern/coping indicator in relation to reported avoidance behaviours. Compared to the reference category (low concern/low coping), high concern/high coping was associated with significantly greater likelihood of avoiding specific locations/events (OR=1.75, p=0.038) and changing overseas travel plans (AOR=1.62, p=0.047), but did not reach statistical significance in relation to changed use of public transport (AOR=1.91, p=0.094).

### Preparedness

Table 3 presents the multivariate findings regarding preparedness behaviours. Reported increases in terrorism-related vigilance were positively associated with: high perceived likelihood of terrorism within Australia (AOR=2.31, p<0.001); high psychological distress (AOR=1.86, p=0.003); and ethnic minority status (AOR=1.98, p=0.005). Terrorism-related learning of evacuation plans was positively associated with female gender (AOR=1.61, p=0.004) and having current paid employment (AOR=5.58, p<0.001). Developing an emergency family contact plan in relation to terrorism concerns was significantly associated with; having children under 16 years of age currently living at home (AOR=1.75, p=0.023) and ethnic minority status (AOR=2.13, p=0.006).

**Table 3:** Terrorism preparedness behaviours by socio-demographic & threat perception variables – adjusted odds ratios (AOR)

**Outcome variable**	**Independent variable**	**Adjusted odd ratios**		
		**AOR**	**[95% CI]**	***p***
**More vigilant for suspicious behaviours**				
	**Terrorist attack likely**			
	No	1.00		
	Yes	**2.31**	**(1.74, 3.06)**	**<0.001**
	**High psychological distress**			
	No	1.00		
	Yes	**1.86**	**(1.23, 2.80)**	**0.003**
	**Ethnic minority status**			
	No	1.00		
	Yes	**1.98**	**(1.23, 3.19)**	**0.005**
**Learned evacuation plan**				
	**Gender**			
	Male	1.00		
	Female	**1.61**	**(1.17, 2.22)**	**0.004**
	**Currently employed**			
	No	1.00		
	Yes	**5.58**	**(3.91, 7.97)**	**<0.001**
**Made emergency family contact plan**				
	**Children in household**			
	No	1.00		
	Yes	**1.75**	**(1.08, 2.83)**	**0.023**
	**Ethnic minority status**			
	No	1.00		
	Yes	**2.13**	**(1.24, 3.64)**	**0.006**

1. Note: 95% confidence intervals (CI) that include 1.00 indicate a non significant result.

2. Independent variables controlled for were: age; highest educational qualification; household income, no. of children ≤ 16 years in household; residential location (urban or rural, and location remoteness via Accessibility/Remoteness Index of Australia (ARIA+); being born in Australia; speaking a language other than English at home (‘minority status’); perceived likelihood of terrorism and self-rated health status, personal resilience (CD-RISC2) and psychological distress (K10).

3. Psychological distress was measured using the K10. Values range from 10–50, with ≥22 considered ‘high’ psychological distress.

**Figure 2:**
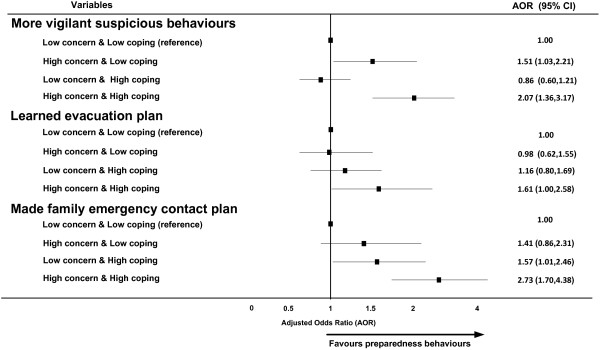
Terrorism related preparedness behaviours by concern/coping combined indicator.

Figure 2 presents the AOR for the concern/coping indicator in relation to reported preparedness behaviours. Compared to low concern/low coping respondents, those with high concern/high coping were significantly more likely to report higher levels of all of the preparedness responses; increased vigilance (AOR=2.07, p=0.001), learning evacuation plans (AOR=1.61, p=0.05) and having made family emergency contact plans (AOR=2.73, p<0.001),

### Evacuation Intentions

The multivariate findings regarding terrorism-related evacuation intentions are presented in Table 4. Greater willingness to evacuate from home showed a highly significant relationship with high income (>$80,000) compared to very low income (<$20,000) (AOR=3.01, p=0.006); and was also positively associated with female gender (AOR=2.01, p=0.012), and high self-rated personal resilience (AOR=1.87, p=0.010). Significantly greater willingness to evacuate from workplaces or public facilities was associated with having children under 16 years living at home (AOR=2.36, p=0.042) and highly accessible residential locations (urban metro) compared to remote/very remote residential location (AOR=0.19, p=0.036). When geographical region replaced ARIA+ in the final model, urban residents reported significantly greater willingness to evacuate from workplaces or public facilities, than rural residents (AOR=0.41, p=0.028).

**Table 4:** Terrorism-related evacuation intentions by socio-demographic & threat perception variables – adjusted odds ratios (AOR)

**Outcome variable**	**Independent variable**	**Adjusted odd ratios**		
		**AOR**	**[95% CI]**	***p***
**Willing evacuate home**				
	**Gender**			
	Male	1.00		
	Female	**2.01**	**(1.16, 3.47)**	**0.012**
	**Household income ($A)**			
	<$20 k	1.00		
	$20-40 k	1.65	(0.78, 3.49)	0.191
	$40-60 k	1.07	(0.47, 2.41)	0.874
	$60-80 k	0.87	(0.45, 1.68)	0.671
	>$80 k	**3.01**	**(1.38, 6.59)**	**0.006**
	**Individual resilience**			
	Low	1.00		
	High	**1.87**	**(1.16, 3.00)**	**0.010**
**Willing evacuate workplace / public facility**				
	**Children in household**			
	No	1.00		
	Yes	**2.36**	**(1.03, 5.38)**	**0.042**
	**Residential location (ARIA+)**			
	Highly accessible (urban)	1.00		
	Accessible	0.51	(0.19, 1.33)	0.166
	Moderately accessible	0.56	(0.21, 1.47)	0.236
	Remote/Very remote	**0.19**	**(0.04, 0.90)**	**0.036**

1. Note: 95% confidence intervals (CI) that include 1.00 indicate a non significant result.

2. Independent variables controlled for were: age; highest educational qualification; household income, no. of children ≤ 16 years in household; residential location (urban or rural, and location remoteness via Accessibility/Remoteness Index of Australia (ARIA+); being born in Australia; speaking a language other than English at home (‘minority status’); perceived likelihood of terrorism and self-rated health status, personal resilience (CD-RISC2) and psychological distress (K10).

3. Psychological distress was measured using the K10. Values range from 10–50, with ≥22 considered ‘high’ psychological distress.

**Figure 3:**
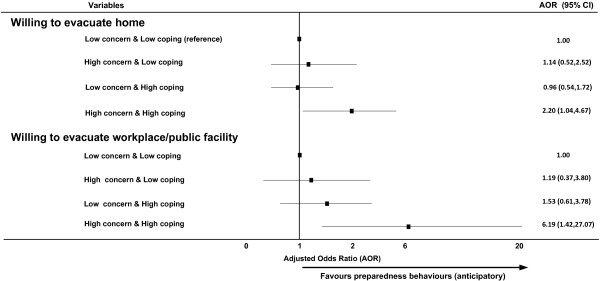
Terrorism related evacuation intentions by concern/coping combined indicator.

Figure 3 presents the AOR for the concern/coping indicator in relation to terrorism-related evacuation intentions. High concern/high coping showed a highly significant relationship with willingness to evacuate from workplaces/public facilities (AOR=6.19, p=0.015), and was also positively associated with willingness to evacuate from home (AOR=2.20, p=0.039).

## Discussion

The current multivariate analysis highlights that terrorism-related avoidance and preparedness behaviours are most consistently associated with cognitive and affective factors, specifically higher concern and perceived coping. The combined concern/coping indicator was positively associated with five of the six current behavioural responses examined in the study and accounted for a broader range of responses than the other cognitive, socio-demographic and health-related factors. Importantly, this combined indicator was associated with all of the current preparedness behaviours as well as evacuation intentions regarding a possible future incident. These findings support other recent data showing that coping self efficacy is strongly associated with terrorism-related preparedness behaviours [14]. Consistent with wider hazard preparedness however, this relationship is mediated by affective factors with higher concern generally being needed to motivate such responses [16,27].

The present findings are consistent with Social-Cognitive models of emergency preparedness [16] but also specific threat- and efficacy-based models such as Witte’s Extended Parallel Process Model. The EPPM addresses the interplay of ‘threat’ and ‘efficacy’ relating to preparedness behaviours and predicts that individuals facing uncertain risks are more likely to engage in proactive behaviours when they regard the threat as legitimate and believe they can perform the required behaviours efficaciously [13]. As such, efficacy perceptions alter the way in which risks are perceived and have been shown to be a key predictor of health worker readiness to respond to major disasters, including terrorism [17,18]. The current data support a similar case regarding general population preparedness for terrorism. High concern/high coping was associated with all of the preparedness behaviours and future intentions, while high concern/low coping predicted increased vigilance alone. As the EPPM and Social-Cognitive models also predict, the combined variables which included ‘low concern’ (e.g. low concern/high coping) were generally not associated with behavioural response. This suggests that high concern may be a necessary (but not sufficient) condition for general population preparedness in this context.

Increased vigilance was the only preparedness behaviour which showed some inconsistency with EPPM, being associated with both high and low coping perceptions. This may be due to the broader range of situations this could entail when compared to the other preparedness responses. Since the first Bali bombing in 2002, the behavioural focus of Australian Government information campaigns has primarily related to increased vigilance (e.g. “Be alert, not alarmed”, “Let’s look out for Australia”) and the reporting of suspicious behaviours to authorities [28]. The current survey item was intended to examine such vigilance behaviours, although the findings may reflect a spectrum of response; from reactive/avoidant coping (characterised by low control/coping) to proactive monitoring and reporting [29]. Recent international campaigns adopted in Australia reflect attempts to direct awareness towards suspicious behaviours (not racial or cultural identifiers) and to operationalise appropriate responses (e.g. “If you see something, say something”) [30]. The greater effect size for vigilance noted with high coping respondents may indicate more proactive monitoring by this group. This prospect could be tested with more detailed research.

Socio-demographic factors such as low education, older age and minority ethnicity may be associated with more reactive/avoidant responses to terrorism threat [31,32]. In this study, low education and low income were among the strongest predictors of avoidance. This supports data from a study in Canada [31]. A possible explanation for these avoidance responses is lack of resources (e.g. financial/physical). Such groups are recognised as key planning partners, [33] and public health emergency planners should work through relevant issues with them to support their preparedness in this context.

Once cognitive and affective factors were accounted for in the present analysis, only three demographic variables independently predicted preparedness: female gender (learned evacuation plan); having dependent children (contact plan); and ethnic minority status (contact plan, vigilance). The importance of establishing family emergency contacts plans was highlighted after the 2005 London bombing [2]. Its current association with parental status is consistent with reported responses of Los Angeles residents one year after the September 11 attacks [34]. Terrorism-related learning of evacuation plans has not previously been found to be associated with gender [31]. This more proactive response in the present findings may relate to care-provider responsibilities or possibly females’ greater general concern with the welfare of others [35]. It is also consistent with the current findings that both female gender and parental status were positive predictors of evacuation willingness. These findings suggest such groups may engage more readily in community risk mitigation efforts for terrorism and possibly act as ‘influence leaders’ for other demographic groups.

It is notable that rural respondents reported being less willing to comply with evacuation requests of non- residential buildings. Although urban areas are presumed to represent higher profile terrorist targets many rural areas house critical infrastructure including potentially vulnerable transport systems and agricultural resources. Rural regions may be the subject of direct terrorist threats requiring infection control, decontamination and evacuation orders [17]. These findings may indicate the need for increased awareness within such communities which contextualises potential threats and details basic preparedness and response requirements.

When considering the implications of the current findings for community preparedness, it is important to note that coping self-efficacy is not defined as a static personal trait, but as competency beliefs that can be altered through experience or education [29]. Recent studies of health workers show that efficacy appraisals are a key factor mediating willingness to respond to major threats, including chemical, biological and radiological (CBR) terrorism [17,18]. Efficacy-focused education for these workers may constitute cognitive ‘interventions’ that promote lower concern and greater response willingness; outcomes that may be crucial in maintaining emergency ‘surge’ capacity [17]. Among Australian paramedics for example, receipt of terrorism-related training focused on key competencies was found to be a stronger predictor of response readiness than career incident experience [36].

Research examining efficacy effects on community preparedness for terrorism remains limited. However, recent evidence indicates that people actively seek efficacy information when facing such threats. Focus groups responding to CBR terrorism scenarios sought: (1) appropriate identification of the risk, and (2) information about concrete actions they could take to increase safety [37]. In practice, personal safety information in pre-event phases is often quite limited (e.g. online pamphlets) [38]. More comprehensive initiatives can be seen in places such as Singapore where citizens can undertake competency based education to enhance their terrorism preparedness. This extends to ‘hands on’ exercises such as the use of ‘In-Place-Protection’ simulators to practice the proper sealing of rooms [39]. While the current data support a relationship between existing efficacy perceptions and preparedness, further research could enhance its real world application by determining: (1) the extent to which personal efficacy appraisals can be raised in this threat context, (2) optimal modalities to achieve this, and (3) and the conditions under which enhanced efficacy appraisals translate to preparedness outcomes.

### Limitations

The current study has several limitations which need to be considered. While the survey response rate of 57.4% compares favourably with similar population studies of this topic, [2] it has the potential to introduce a response bias in relation to the current results. This issue was addressed by introducing weightings to adjust for probability of selection and for differing response rates among males and females and different age groups.

The primary aim of this study was to examine the relationship between terrorism concern/coping indicators and preparedness for terrorism using a relatively large, State-based sample. While this New South Wales data set is representative of the national population in terms of its key demographic characteristics [26], potential regional differences regarding perceived terrorism threat and response means that the findings may not generalise to all Australian States. Response bias may also have been associated with the use of telephone interviews and conducting the survey in English. Although the sample size is a strength of this study, its cross-sectional design presents responses at a single time point and no firm conclusions can be made regarding causes. The final question set is not a comprehensive list of all the possible behaviours individuals could undertake in response to terrorism threat, although it was thought to reflect relevant responses in the Australian threat context. Similarly, the evacuation items reflect intentions only and may not be predictive of behaviour in a response situation. This exploratory analysis only examined a subset of possible predictors of behavioural responses in this context and cannot preclude the possible contribution of other factors which were not measured in this study.

## Conclusion

Practical preparedness activities related to terrorism threats are consistently associated with personal coping appraisals, while also being mediated by levels of personal concern. In contrast, socio-demographic and other cognitive/affective factors were found to be less predictive of such preparedness. Perceived individual coping with terrorism, at both general and incident-specific levels, represent viable intervention targets as part of population preparedness initiatives and may support broader community adaptation to this threat.

## Competing interests

The authors declare that they have no competing interests.

## Authors’ contributions

GS and MT conceived the idea and designed the study. GS carried out the statistical analysis and wrote the manuscript. All authors made contributions to the interpretation of results and revised the manuscript for important intellectual content. All authors read and approved the final version of the manuscript.

## Pre-publication history

The pre-publication history for this paper can be accessed here:

http://www.biomedcentral.com/1471-2458/12/1117/prepub
